# A rare case of P-ANCA-associated endomyocardial fibrosis without eosinophilia

**DOI:** 10.1093/ehjcr/ytag289

**Published:** 2026-04-25

**Authors:** Khurram Butt, Fox Bravo, Amitoj Singh, Tushar Acharya

**Affiliations:** Sarver Heart Center, University of Arizona, Tucson, 1501 N Campbell Ave, Tucson, AZ 85724, USA; College of Medicine, University of Arizona, 1501 North Campbell Avenue, Tucson, AZ 85724, USA; Sarver Heart Center, University of Arizona, Tucson, 1501 N Campbell Ave, Tucson, AZ 85724, USA; Sarver Heart Center, University of Arizona, Tucson, 1501 N Campbell Ave, Tucson, AZ 85724, USA

## Case description

**Figure 1:**
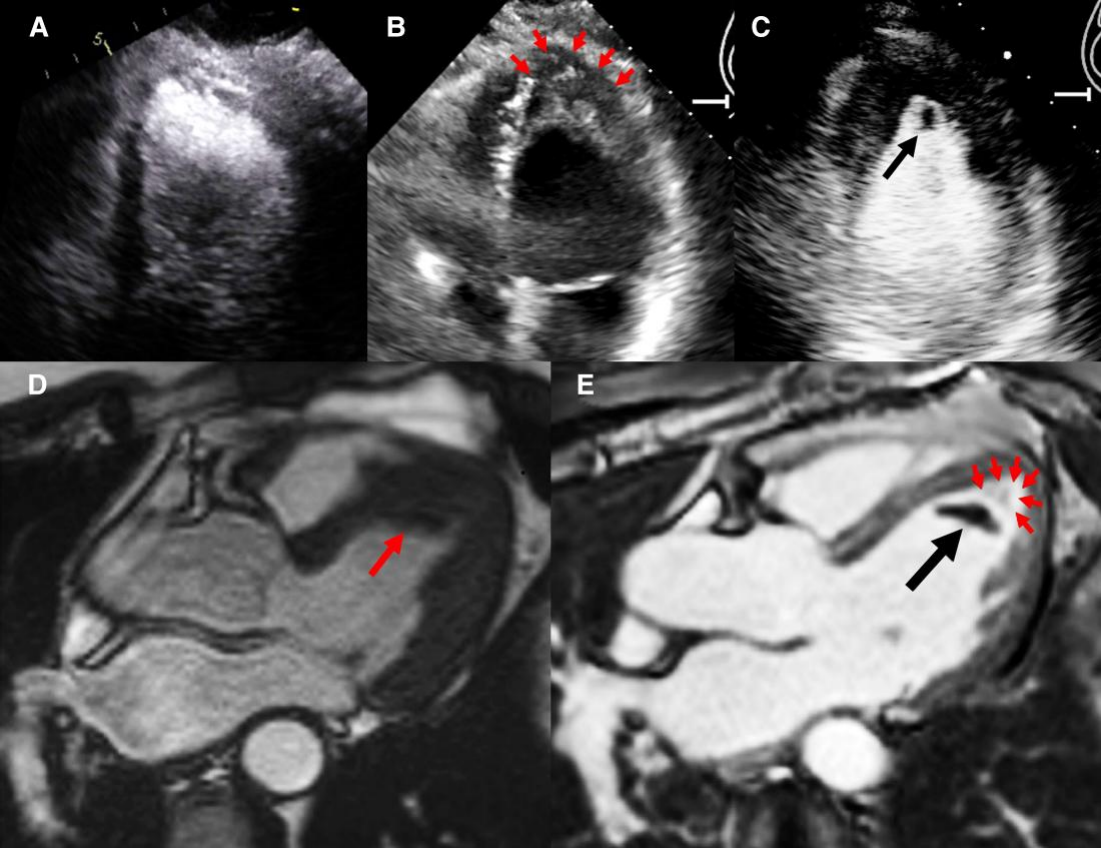
Multimodal cardiac imaging demonstrating development of endomyocardial fibrosis. Red arrows indicate endomyocardial fibrosis/thickening; black arrows indicate LV apical thrombus. (*A*) Contrast-enhanced TTE obtained 3 years prior to current presentation showing normal LV apex without endomyocardial thickening or thrombus, with normal LVEF and wall motion. (*B*) Repeat TTE without contrast at the current presentation showing increased apical wall thickness (red arrows). (*C*) Contrast-enhanced TTE confirming apical thrombus (black arrow). (*D*) Cardiac MRI cine confirming thickening of apical segments (red arrow). (*E*) Late gadolinium enhancement imaging showing dense fibrosis (red arrows) in the endomyocardial layer of the apical segments and confirming LV apical thrombus (black arrow).

A 76-year-old man with biopsy-proven P-ANCA-associated pauci-immune glomerulonephritis and interstitial lung fibrosis since 2021, on interrupted immunosuppression, was admitted for slurred speech from a transient ischaemic attack in 2025. Echocardiography for cardioembolic evaluation revealed preserved left ventricular ejection fraction but significant apical wall thickening and an apical thrombus (*Figure 1B* and *C*). Coronaries were normal on invasive angiography.

Subsequently, CMR for further evaluation demonstrated marked wall thickening with cavity obliteration in the apical segments. Late gadolinium enhancement (LGE) had the typical three-layered pattern of normal myocardium, thickened enhanced endomyocardium representing fibrosis (*Figure 1D*), and overlying non-enhancing apical thrombus (*Figure 1E*).^1^ Contrast echocardiogram in 2022 (3 years prior to current presentation) was notable for the absence of apical wall thickening or thrombus (*Figure 1A*), suggesting a more recent endomyocardial injury.

The differential included Loeffler endocarditis, apical hypertrophic cardiomyopathy, cardiac sarcoidosis, and thrombus from prior infarction. The absence of eosinophilia, a characteristic LGE three-layered pattern, and normal coronary arteries favoured ANCA-associated EMF in this case.

Notably, the patient did not have a history of asthma. Although eosinophilia in ANCA-associated vasculitis may be transient, serial blood counts with differential obtained daily over 14 days of hospitalization and on outpatient visits obtained over 3 years did not demonstrate peripheral eosinophilia. Prior renal biopsy did not reveal eosinophilic infiltrates either. These findings argue against EGPA and suggest an eosinophil-independent pathway of P-ANCA-associated myocardial injury. The precise mechanism by which P-ANCA-associated vasculitis causes myocardial injury is unknown. One potential mechanism involves ANCA-activated neutrophils causing endothelial injury via degranulation, reactive oxygen species, and neutrophil extracellular trap formation.^2^ The patient was initiated on systemic anticoagulation and referred for immunosuppressive therapy.

This case highlights CMR’s role in detecting subclinical myocardial involvement in ANCA-associated vasculitis^3^ and underscores the possibility of developing EMF in the absence of typical eosinophilic features. Early identification of EMF allows for appropriate anticoagulation to prevent thromboembolic complications and guide immunosuppressive management.

## Data Availability

The data underlying this article cannot be shared publicly to protect the privacy of the patient. Deidentified data may be made available to qualified researchers upon reasonable request to the corresponding author.

## Notes

**Consent**: has been obtained from the patient.
